# Augmentation of Rotator Cuff Repair With a Bioinductive Collagen Scaffold: The Arthroscopic Slide Technique

**DOI:** 10.1002/atn2.70286

**Published:** 2026-10-09

**Authors:** Peter A. Bueide, Jordan D. Shearer, Ryan M. Peldo, Nathan W. Skelley

**Affiliations:** ^1^ Department of Graduate Medical Education Sanford Health Fargo North Dakota U.S.A.; ^2^ School of Medicine & Health Sciences University of North Dakota Grand Forks North Dakota U.S.A.; ^3^ University of South Dakota Sanford School of Medicine Sioux Falls South Dakota U.S.A.

## Abstract

Rotator cuff repair continues to be one of the most common orthopaedic surgeries performed. However, depending on patient risk factors, retear rates remain high. We describe a technique for tensioning a collagen scaffold to supplement rotator cuff repair using laterally based pulley sutures. This ensures the scaffold is fixed in appropriate tension to function as a load‐sharing construct with rotator cuff repair. Aside from the biologic scaffold, this technique requires minimal extra implant costs except for 2 additional sutures.

## Graphical abstract

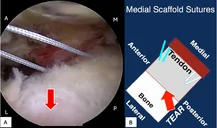

**VIDEO 1:** Arthroscopic rotator cuff repair with BioBrace (Conmed, Largo, FL) bioinductive collagen scaffold. A standard double‐row rotator cuff repair is performed, and the scaffold is fixed medially using 2 prepositioned number 2, high‐tensile, Hi‐Fi (Conmed) inverted horizontal mattress sutures. The scaffold is fixed laterally using the preloaded sutures in the lateral‐row Argo (Conmed) biocomposite rotator cuff anchors to create a pulley mechanism over the lateral fixation sutures in the scaffold. This allows the scaffold to be tensioned over top of the repair and secured without a third row of anchors. Video content can be viewed at https://doi.org/10.1002/atn2.70286.

A rotator cuff tear (RCT) is a common condition treated by orthopaedic surgeons. The prevalence of RCTs ranges widely, with patients older than 80 years reporting prevalence of 62% or higher.^1^ Many patients have degenerative RCTs on advanced imaging; however, longitudinal studies have found many of these patients are asymptomatic and remain so for an extended period of time.^2^ When symptomatic, many degenerative RCTs are successfully managed conservatively as a first‐line treatment.^3^, ^4^ However, for patients with persistent pain, weakness, or functional limitation despite nonoperative care, rotator cuff repair (RCR) remains a viable option.^5^, ^6^ In the setting of acute, traumatic RCTs, surgical intervention is considered by some a first‐line strategy owing to its superior clinical outcomes.^7^, ^8^, ^9^ Retear of RCR surgeries continues to be a challenging problem for surgeons and patients.^10^, ^11^, ^12^

A risk calculator (Rotator Cuff Healing Index score) has been developed in an attempt to identify patients who are at risk of RCR failure based on age greater than 70 years, anteroposterior tear size greater than 2.5 cm, length of medial‐to‐lateral tear retraction, fatty infiltration greater than Goutallier grade II, osteoporosis with *t* score less than −2.5, and high level of work.^10^ Other factors including age, sex, prior RCR, and Workers’ Compensation claims have also been found to be risk factors for RCR failure.^11^, ^13^, ^14^, ^15^

Bioinductive implant augmentation has emerged as a strategy to improve retear rates and patient‐reported outcomes, as evidenced by the most recent American Academy of Orthopaedic Surgeons clinical practice guideline on RCR.^16^ A range of graft materials and fixation strategies have been described.^17^, ^18^, ^19^, ^20^ Bioinductive collagen patches appear to be safe and promote tendon healing across numerous studies, with potentially lower retear rates and good clinical outcomes.^19^, ^21^, ^22^, ^23^ Furthermore, patches that have mechanical strength properties can also function as load‐sharing devices that protect a RCR.^24^ The technique described in this article—the arthroscopic slide—is an arthroscopic double‐row RCR with scaffold augmentation. The technique emphasizes independent scaffold fixation using lateral pulley sutures, permitting adjustable tensioning of the augment without disturbing the RCR, and creates a load‐sharing construct (Video 1).

## SURGICAL TECHNIQUE

### Patient Indications

Patients are indicated for RCR with BioBrace (Conmed, Largo, FL) collagen scaffold augmentation if they have a full‐ or partial‐thickness rotator cuff that is atraumatic in nature and they have risk factors for retear including chronic tear, increased age, poor intraoperative tissue quality, fatty atrophy noted on preoperative magnetic resonance imaging, and large tear size. Patients are consented preoperatively regarding the possibility of augmenting their RCR.

### RCR and Medial Scaffold Preparation

Prior to beginning RCR, a standard diagnostic arthroscope of the shoulder is performed. The RCT is identified and debrided. The tear is evaluated for tissue size, tissue quality, and degree of retraction, and the decision to proceed with augmentation is made. Using an arthroscopic suture passer device, 2 number 2 Hi‐Fi (Conmed) high‐tensile strength sutures of different color are passed in an inverted horizontal mattress fashion ~1.5 cm apart at the anterior and posterior aspect of the RCT, medial to the location of the medial‐row sutures of the RCR. It is important that the suture limbs exit on the superior aspect of the rotator cuff, in the subacromial space (Figure 1). These suture limbs are then retrieved through accessory portals anteriorly and posteriorly and clamped to the sterile drapes for suture management during RCR.

**FIGURE 1:**
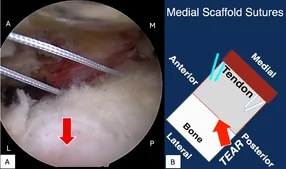
(A) Arthroscopic view from the posterolateral viewing portal of a left shoulder of a medial inverted number 2, high‐tensile, Hi‐Fi (Conmed, Largo, FL) horizontal mattress suture placed in the anterior rotator cuff medial and, prior, to the future rotator cuff repair sutures. A second suture will be placed in the posterior rotator cuff. These sutures will be used to fix the medial scaffold after rotator cuff repair is complete. (B) Illustration showing the anterior and posterior inverted horizontal mattress sutures for medial scaffold fixation. Arrow points to rotator cuff tear. (A, anterior; L, lateral; M, medial; P, posterior.)

The primary operating surgeon in this study prefers a double‐row, medial‐row knotted, and lateral‐row knotless repair. Triple‐loaded CrossFT (Conmed) biocomposite suture anchors are used for medial‐row anchors and fixation. These are placed just lateral to the articular margin of the humeral head in an appropriate anterior‐posterior position relative to the specific tear pattern and size. The sutures are passed and tied to complete the medial‐row repair (Figure 2). The lateral row is then secured in a crisscrossing double‐row construct using knotless preloaded Argo biocomposite anchors (Conmed) (Figure 3). The RCR suture tails are then cut with an arthroscopic cutter. The preloaded free suture in the lateral‐row Argo anchor remains for fixation of the BioBrace laterally. These preloaded Argo suture limbs are then retrieved through the lateral working portal outside of the cannula. A small nick is made in the side of the silicone cannula outer ring for these sutures to be temporarily cleated and secured to prevent twists or tangles in the lines during the BioBrace deployment.

**FIGURE 2:**
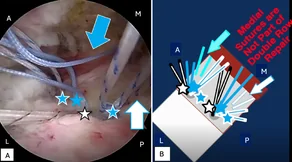
(A) Arthroscopic view from the posterolateral viewing portal of a left shoulder of the medial number 2, high‐tensile, Hi‐Fi (Conmed, Largo, FL) inverted horizontal mattress sutures (arrows), medial to the medial‐row rotator cuff repair sutures. Stars represent location of rotator cuff repair knots; the 2 posterior knots have been tied in the image. (B) Illustration showing the anterior and posterior inverted horizontal mattress sutures for medial scaffold fixation, medial and separate from the tied medial‐row rotator cuff repair sutures (stars represent knots). (A, anterior; L, lateral; M, medial; P, posterior.)

**FIGURE 3:**
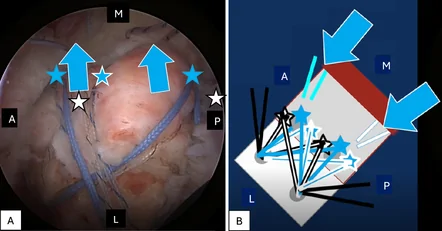
(A) Arthroscopic view of a left shoulder from a lateral viewing portal of the medial number 2, high‐tensile, Hi‐Fi (Conmed, Largo, FL) inverted horizontal mattress sutures (arrows), medial to the completed double‐row rotator cuff repair. Stars represent location of medial‐row rotator cuff repair knots. (B) Illustration showing the Hi‐Fi inverted horizontal mattress sutures (arrows) for medial scaffold fixation, medial and separate from the completed double‐row rotator cuff repair (stars represent knots). Note the additional 2 suture limbs from each of the lateral‐row Argo (Conmed) biocomposite anchors (black lines). (A, anterior; L, lateral; M, medial; P, posterior.)

### Scaffold Deployment and Medial Fixation

The BioBrace augment is loaded using the preattached BioBrace RC Delivery System according to the manufacturers’ surgical technique guide. The horizontal mattress sutures placed medial to the RCR at the beginning of the procedure are unclamped from the drapes and consecutively retrieved from the lateral working portal and loaded through the scaffold medially using the BioBrace RC Delivery System (Conmed) in the appropriate positions. Alternatively, these can be passed with a free needle or arthroscopic suture passer of choice if the surgeon is using a BioBrace alone and not the BioBrace RC Delivery System. The BioBrace is then delivered through the lateral working cannula and slid down the medial fixation sutures while holding tension on these sutures to avoid tangling or twists within the lines (Figure 4). The medial horizontal mattress sutures are then consecutively retrieved from the accessory portals, tied, and cut. In this retrieval process, care is taken to use a suture retriever device and capture both limbs of the same suture and pull these limbs out of the accessory portal before tying and securing the BioBrace medially. This process prevents any tissue bridges if the surgeon is tying through the accessory portal without a cannula. This process is repeated for the other medial suture. The preloaded retention suture is then removed from the BioBrace RC scaffold if the delivery system is used.

**FIGURE 4:**
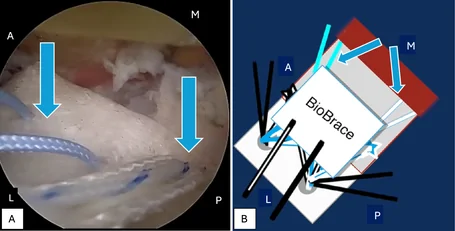
(A) Arthroscopic view of a left shoulder shows the BioBrace (Conmed, Largo, FL) scaffold augment from the posterolateral portal, laying on top of the complete double‐row rotator cuff repair, with the medial number 2, high‐tensile, Hi‐Fi (Conmed) inverted horizontal mattress sutures passing through the medial aspect of the scaffold (arrows). (B) Illustration showing the scaffold passed along the inverted horizontal mattress sutures (arrows), on top of the completed rotator cuff repair. (A, anterior; L, lateral; M, medial; P, posterior.)

### Lateral Fixation Technique of RCR Scaffold

The BioBrace RC scaffold comes preloaded with lateral luggage tag fixation sutures. If a non‐preloaded scaffold is used, appropriately spaced luggage sutures will need to be placed in the lateral aspect of the augment. The anterior Argo anchor preloaded sutures are then uncleated from the lateral cannula. The anterior Argo sutures and anterior BioBrace lateral fixation luggage tag suture are then removed from the lateral cannula. A loop grasper is used to retrieve the anterior lateral BioBrace fixation suture between both limbs of the Argo sutures. An arthroscopic knot pusher is used to tie 2 alternating half hitches with the Argo sutures over the lateral BioBrace suture. This lateral suture is held by an assistant with gentle tension. In this process, a knot pulley is created on top of the anchor with the lateral BioBrace suture passing under this knot (Figure 5). The arthroscopic slide technique is then performed by pulling on the lateral BioBrace scaffold suture to obtain the ideal tension and load‐sharing fixation. The repair‐scaffold construct is observed with the camera as the BioBrace suture limbs are tensioned to the desired level. The lateral suture is then cross‐tied into the existing Argo suture knot from the pulley mechanism with 4 to 5 half hitches. This process is repeated for the posterior lateral fixation point using the same technique. Suture tails are then cut and the repair‐scaffold construct is inspected (Figure 6).

**FIGURE 5:**
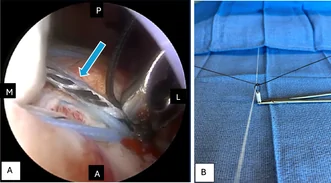
(A) Arthroscopic view of a left shoulder from an anterolateral portal shows the arthroscopic slide technique. The lateral Argo (Conmed, Largo, FL) biocomposite anchor suture is tied over the lateral scaffold suture (arrow), essentially creating a pulley for tensioning the scaffold augment. (B) Ex vivo illustration of the arthroscopic slide pulley technique. The scaffold suture is passed between the 2 limbs of the lateral‐row Argo (Conmed) biocomposite anchor suture, which is tied with 2 alternating half hitches over the scaffold suture. Note the knot is not tensioned in the figure to show the pulley loop. This effectively creates a pulley to lateralize the scaffold to the desired tension. The lateral scaffold suture will serve as the post once the desired tension is placed on the scaffold augment and is tied into the pulley stitches for definitive fixation. (A, anterior; L, lateral; M, medial; P, posterior.)

**FIGURE 6:**
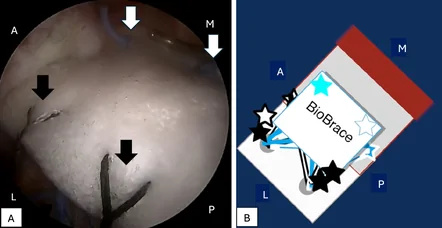
(A) Arthroscopic view of the completed BioBrace (Conmed, Largo, FL) scaffold augment from a posterolateral portal after fixation. White arrows show knots of the medial horizontal mattress sutures; black arrows point to lateral scaffold sutures. Note tension on the scaffold adjacent to the lateral scaffold sutures. (B) Illustration of the complete repair (stars represent knots). (A, anterior; L, lateral; M, medial; P, posterior.)

## DISCUSSION

We describe a technique for augmented RCR, the “arthroscopic slide.” We find this technique to be useful in patients at risk of retear, including older patients with large, degenerative tears and poor tissue quality.^10^, ^11^, ^13^, ^14^, ^15^ This technique allows for double‐row RCR with independent fixation of a bioinductive scaffold. It does not require additional implants to a standard double‐row repair aside from the scaffold and high‐tensile sutures, 2 medial to the RCR construct and 2 in the lateral augment. Scaffold‐augmented RCR increases the implant cost of the procedure. However, when scaffold augments are otherwise indicated, this technique avoids the extra cost associated with an additional third row of anchors and still achieves the surgical goals of biologic augmentation with a load‐sharing mechanical construct. This also minimizes the loss of native bone with additional anchors. Although we used a BioBrace scaffold and CrossFT and Argo anchors, this technique could be modified for any other anchor configuration. The pulley suture allows the surgeon to lateralize the construct and control the desired tension, maximizing interface of the scaffold with the repair and theoretically improving biologic integration. Furthermore, we place tension on the construct with this lateral fixation method, which creates the load‐sharing benefits of the scaffold.^24^ Lastly, this technique could be used in other operative areas where controlled tension on a graft or scaffold is desired. Advantages and disadvantages of this technique are outlined in Table 1.

**TABLE 1:** Advantages and Disadvantages of Scaffold‐Augmented RCR Using the “Arthroscopic Slide”

**Advantages**	**Disadvantages**
Avoids the cost of extra anchors compared with other techniques relying on a third row of anchors	Scaffold augments add cost to traditional RCR
Preservation of native bone stock by avoiding additional suture anchors	Scaffold augments add suture burden and require meticulous suture management
Staged suture management minimizes scaffold entanglement with RCR sutures, which are cut by the time the scaffold is implanted	Scaffold augments add time to traditional RCR
Modifiable for any scaffold and implant system	Relies on knot tying for both medial and lateral fixation of the scaffold
“Arthroscopic slide” tensioning could be used in other anatomic locations requiring controlled tension	Spacing of fixation points is critical to obtain appropriate tension and coverage
Allows adjustable tension on scaffold construct	

RCR, rotator cuff repair.

There are an array of scaffolds and techniques available to the surgeon for augmenting RCR, which will only be expected to increase as tissue scaffolds are further studied and implemented into practice.^17^, ^18^, ^19^, ^20^ Current American Academy of Orthopaedic Surgeons guidelines support surgeons’ incorporation of augmented RCR techniques to treat these complex tears.^16^ We find this technique to be technically satisfying. It adheres to the principles of creating a load‐sharing construct with biologic augmentation, and it is facile to complete with practice.

### Pearls and Pitfalls

Pearls and pitfalls of this technique are outlined in Table 2. First, ensure the medial horizontal mattress sutures are appropriately spaced relative to the tear size and the scaffold to ensure the scaffold lays flat over the tear. We have found a 1.5‐cm spacing between sutures to be optimal. The width of the suture passing device can be used to facilitate this measurement and spacing process. Second, using different suture colors for the medial scaffold horizontal mattress sutures aids in suture management and efficient scaffold fixation later in the case. Third, the lateral‐row anchor sutures are outside the portal cannula during scaffold placement to prevent tangling with the medial sutures as the scaffold is passed and fixed. Fourth, any time knots are tied without a cannula; the suture tails must be obtained together with a retriever device to prevent any soft‐tissue bridges. Alternative suture management strategies that permit only tying through a cannula may avoid this issue as well. Finally, in the event the lateral pulley fixation fails; a third row of anchors may be used to complete the repair.

**TABLE 2:** Pearls and Pitfalls of Scaffold‐Augmented RCR Using the “Arthroscopic Slide”

**Pearls**	**Pitfalls**
1.5‐cm spacing of medial horizontal mattress sutures appropriately flattens the scaffold on final tensioning	Always withdraw suture tails simultaneously from noncannulated portals to avoid suture bridges with knot tying
Use different colors for medial horizontal mattress sutures to aid in suture management when fixing the scaffold	The medial horizontal mattress sutures should be managed meticulously during RCR, or else risk tangling with RCR sutures throughout the case
Removing the lateral‐row sutures outside the lateral cannula until needed for lateral scaffold fixation avoids inadvertent tangles with the medial horizontal sutures being passed for scaffold fixation	When passing medial horizontal mattress sutures through the scaffold, care should be taken to avoid twists in the suture limbs, ensuring knots hold the scaffold flush to the underlying repair
Ensure the lateral scaffold suture is the post when initially tying to the lateral anchor sutures in the “arthroscopic slide” knot to optimally tension the construct	When tying the “arthroscopic slide” knot, lack of direct visualization of the knot with the camera risks slack in the repair if incompletely tensioned
A third row of anchors can be used laterally if fixation with the lateral‐row anchors fails for any reason	Care should be taken to protect and not inadvertently remove the medial‐row fixation sutures, or it will complicate fixation across the 4 points

RCR, rotator cuff repair.

## DISCLOSURES

The author (N.W.S.) declares the following financial interests/personal relationships which may be considered as potential competing interests: N.W.S. was a consultant to Conmed on a device unrelated to this publication and has received travel and education support from Arthrex and Stryker. The other authors (P.A.B., J.D.S., R.M.P.) declare that they have no known competing financial interests or personal relationships that could have appeared to influence the work reported in this article.
